# Hyaluronic Acid-Based Nanoparticles Loaded with Rutin as Vasculo-Protective Tools against Anthracycline-Induced Endothelial Damages

**DOI:** 10.3390/pharmaceutics16080985

**Published:** 2024-07-25

**Authors:** Carla Serri, Vincenzo Quagliariello, Iriczalli Cruz-Maya, Vincenzo Guarino, Nicola Maurea, Paolo Giunchedi, Giovanna Rassu, Elisabetta Gavini

**Affiliations:** 1 Department of Medicine, Surgery and Pharmacy, University of Sassari, Via Muroni 23/A, 07100 Sassari, Italy; cserri@uniss.it (C.S.); pgiunc@uniss.it (P.G.); eligav@uniss.it (E.G.); 2 Division of Cardiology, Istituto Nazionale Tumori-IRCCS-Fondazione G. Pascale, 80131 Naples, Italy; quagliariello.enzo@gmail.com (V.Q.); n.maurea@istitutotumori.na.it (N.M.); 3 Institute of Polymers, Composites and Biomaterials, National Research Council of Italy, Mostra d’Oltremare Pad. 20, V.le J.F. Kennedy 54, 80125 Naples, Italy; cdiriczalli@gmail.com (I.C.-M.); vincenzo.guarino@cnr.it (V.G.)

**Keywords:** Rutin, hybrid nanoparticles, hyaluronic acid, anthracyclines, cancer

## Abstract

Anthracycline-based therapies exert endothelial damages through peroxidation and the production of proinflammatory cytokines, resulting in a high risk of cardiovascular complications in cancer patients. Hyaluronic acid-based hybrid nanoparticles (LicpHA) are effective pharmacological tools that can target endothelial cells and deliver drugs or nutraceuticals. This study aimed to prepared and characterized a novel LicpHA loaded with Rutin (LicpHA Rutin), a flavonoid with high antioxidant and anti-inflammatory properties, to protect endothelial cells against epirubicin-mediated endothelial damages. LicpHA Rutin was prepared using phosphatidylcholine, cholesterol, poloxamers, and hyaluronic acid by a modified nanoprecipitation technique. The chemical-physical characterization of the nanoparticles was carried out (size, zeta potential, morphology, stability, thermal analysis, and encapsulation efficiency). Cytotoxicity studies were performed in human endothelial cells exposed to epirubicin alone or in combination with Free-Rutin or LicpHA Rutin. Anti-inflammatory studies were performed through the intracellular quantification of NLRP-3, MyD-88, IL-1β, IL-6, IL17-α, TNF-α, IL-10, and IL-4 using selective ELISA methods. Morphological studies via TEM and image analysis highlighted a heterogeneous population of LicpHA particles with non-spherical shapes (circularity equal to 0.78 ± 0.14), and the particle size was slightly affected by Rutin entrapment (the mean diameter varied from 179 ± 4 nm to 209 ± 4 nm). Thermal analysis and zeta potential analyses confirmed the influence of Rutin on the chemical-physical properties of LicpHA Rutin, mainly indicated by the decrease in the surface negative charge (from −35 ± 1 mV to −30 ± 0.5 mV). Cellular studies demonstrated that LicpHA Rutin significantly reduced cell death and inflammation when compared to epirubicin alone. The levels of intracellular NLRP3, Myd-88, and proinflammatory cytokines were significantly lower in epirubicin + LicpHA Rutin-exposed cells when compared to epirubicin groups (*p* < 0.001). Hyaluronic acid-based nanoparticles loaded with Rutin exerts significant vasculo-protective properties during exposure to anthracyclines. The overall picture of this study pushes towards preclinical and clinical studies in models of anthracycline-induced vascular damages.

## 1. Introduction

Anthracyclines, anticancer drugs composed of epirubicin, doxorubicin, and idarubicin, are currently clinically used to treat patients with breast cancer, lymphomas, leukaemia, and head and neck tumours. However, well-known dose-dependent cardiotoxicity, including heart failure and cardiomyopathies, are frequently seen in clinical scenarios [1]. Cardiotoxic events in cancer patients treated with anthracyclines were described in 5% and up to 48% of patients (when treated with a cumulative dose of 400 mg/m^2^ and 700 mg/m^2^, respectively), affecting their prognosis and overall survival [2]. Very recently, endothelial inflammation was described as the key orchestrator of epirubicin-induced cardiac toxicity [3]. Notably, endothelial cells damaged by doxorubicin and epirubicin can trigger the development and progression of tissue inflammation through the reduction of protective endothelial factors (including prostaglandin type I 2, zonulin, and nitric oxide) [4]. Clinical evidence also describes endothelial-mediated cognitive impairments induced by epirubicin and doxorubicin in patients with cancer [5]. Mechanistically, epirubicin induced tissue side effects, primarily in endothelial cells, through the induction of ferroptosis, necrosis, inflammation, and oxidation-mediated cell damages [6,7]. These events are the first initiators of epirubicin-mediated cardiovascular damages; therefore, the endothelium represents a novel target of cardiovascular protection in patients with cancer.

New pharmacological therapies, including nanoparticles targeting endothelial cells and those loaded with antioxidant and anti-inflammatory bioactives, could be strictly needed in oncology settings to improve cancer patients’ quality of life and reduce cardiac and vascular events in these patients [8]. In recent years, flavonoids have been used for their beneficial pharmacological effects and safety. Indeed, the antioxidant properties of flavonoids, largely related to their chemical structure and reducing specifications, are responsible for various therapeutic effects, such as cardiovascular damage [9]. Rutin, also known as quercetin-3-rutinoside or sophorina, is a natural polyphenolic bioflavonoid derivative with anti-inflammatory and scavenging properties against oxidative species. Rutin is naturally present in vegetables, citrus fruits, and various plant species belonging to the Primulaceae, Rutaceae, Myrtaceae, and Cannaceae family [10]. Several recent preclinical and clinical studies have shown that Rutin has antibacterial, antiviral, cardioprotective, neuroprotective, and nephroprotective effects [11,12,13,14]. Nevertheless, when orally administered, Rutin has low bioavailability due to poor water solubility, poor absorption in the small intestine, and metabolism by intestinal bacteria and the liver (hepatic first-pass metabolism) [15,16]. Different nanotechnology approaches, particularly polymeric and lipid nanoparticles, have been studied to improve the solubility, pharmacokinetic properties, and, thus, efficacy of Rutin [10]. Among them, only Ahmad and collaborators explored the benefits of the intranasal administration of Rutin encapsulated in chitosan nanoparticles on the pharmacokinetics of Rutin [17].

Based on these assumptions, this work aimed to prepare and characterize Rutin-loaded hybrid nanoparticles (LicpHA Rutin) to treat inflammation induced by epirubicin, a model anthracycline, in endothelial cells.

Herein, polymer–lipid hybrid nanoparticles were selected to merge the advantages of liposomes and polymeric nanoparticles as drug carriers, overcoming their main limitations (i.e., low circulatory half-lives due to absorption by the reticuloendothelial system (RES), extended burst kinetics due to exceeding drug loads on the surface) [18]. In particular, the peculiar two-in-one structure shows improved physicochemical stability and increased biocompatibility [19]. In the literature, other lipid–polymer hybrid nanoparticles loaded with Rutin have been reported. Ishak and co-workers prepared hybrid nanoparticles composed of a poly(lactic-co-glycolic acid) (PLGA) core and a phospholipid shell entangled with PEG-based surfactants [20]. Similarly, Julio and collaborators studied hybrid nanoparticles based on choline-based ionic liquids and PLGA [21]. Herein, for the first time, hybrid nanoparticles (LicpHA) were formulated as carriers of Rutin using phosphatidylcholine (LP) and hyaluronic acid (HA) with a poloxamer. HA is an attractive ligand for inflammation-tumour targeting and is suitable for delivering drugs in the presence of inflammation and cancer. In particular, HA is biocompatible, non-immunogenic, and can specifically bind CD44 and receptors for mediated motility (RHAMMs), which are overexpressed in many inflammatory cells [22,23,24]. HA is a biodegradable polymer widely used in drug delivery for mucoadhesive and penetration-enhancing properties [25]. Poloxamer (P), an amphiphilic copolymer based on one block of poly(propylene oxide) (PPO) and two blocks of poly(-ethylene oxide) (PEO), was chosen due to its ability to interact with phospholipids of the membrane bilayer and to repair damaged biological membranes, and also because of its non-toxicity, safety, and applicability [26,27]. In particular, when used alone, Poloxamer 407 has no relevant advantages; when combined with mucoadhesive polymers, P promotes mucoadhesion [28,29]. Moreover, poloxamers bind HA chains as ‘bridging’ molecules, creating a cross-linked system that provides strength and drug-loading capacity [22,30].

LicpHA and LicpHA Rutin were characterized in terms of size, surface charge, total drug in dispersion, encapsulation efficiency, in vitro Rutin release, and morphology; differential scanning calorimetry was used to provide additional information on the structure and properties of the nanoparticles. The physical stability of the LicpHA and LicpHA Rutin in dispersion was evaluated in terms of time, temperature, and dilution in the RPMI-1640 cell medium. Considering the clinically relevant neurotoxicity and endothelial dysfunctions in cancer patients treated with anthracyclines [31], the potential cytoprotective effects of LicpHA Rutin on in vitro models of endothelial cells exposed to clinically relevant doses of epirubicin were tested. In detail, in vitro cell studies were performed on human umbilical vein endothelial cells (HUVECs) as a model of endothelial cells to evaluate the cytoprotective effects of hybrid nanoparticles and their cellular uptake. Considering that anthracyclines induce a pro-inflammatory phenotype in endothelial cells through the overexpression of the NLRP-3 (inflammasome) and MyD-88 (myddosome) pathways [32], which in turn induce the release of chemokines, growth factors, IL-1β, and IL-6, the intracellular levels of these factors were measured after exposure to epirubicin in the presence of LicpHA Rutin.

## 2. Materials and Methods

### 2.1. Materials

Rutin hydrate (Quercetin-3-rutinoside hydrate, Vitamin P hydrate) (purity > 94%, water solubility: 0.125 g/L), cholesterol (Chol), and Poloxamer F407 (P) (Poly (ethylene glycol)-block-poly (propylene glycol)-block poly (ethylene glycol)) were purchased by Sigma-Aldrich from Merck (Milan, Italy). Lipoid S 100 was gifted by a gift from Lipoid GmbH (Ludwigshafen, Germany) (LP), and hyaluronic acid sodium salt (HA), with a molecular weight of 799.74 kDa, from BioChemical-Fluka (Milan, Italy). Dimethyl sulfoxide (DMSO), ethanol, Na_2_HPO_4_, NaCl, KCl, and Nile Red (NR) were obtained from Sigma-Aldrich (Milan, Italy). The Roswell Park Memorial Institute (RPMI-1640) medium and human endothelial cells (HUVEC cell line) were obtained from the American Type Culture Collection (Manassas, VA, USA) and were cultured in Dulbecco’s modified Eagle’s medium/F12 (Sigma, Merck KGaA, Darmstadt, Germany) supplemented with 10% (*v*/*v*) of foetal bovine serum (FBS) (Microtech, Milan, Italy) and 1% (*v*/*v*) penicillin/streptomycin (Microtech, Milan, Italy) at 37 °C in a humidified 5% CO_2_ atmosphere. The pure water was prepared by a Milli-Q R4 system (Millipore, Milan, Italy). All chemicals and media were used as received without any further purification.

### 2.2. Preparation of Hybrid Nanoparticles

**Figure 1:**
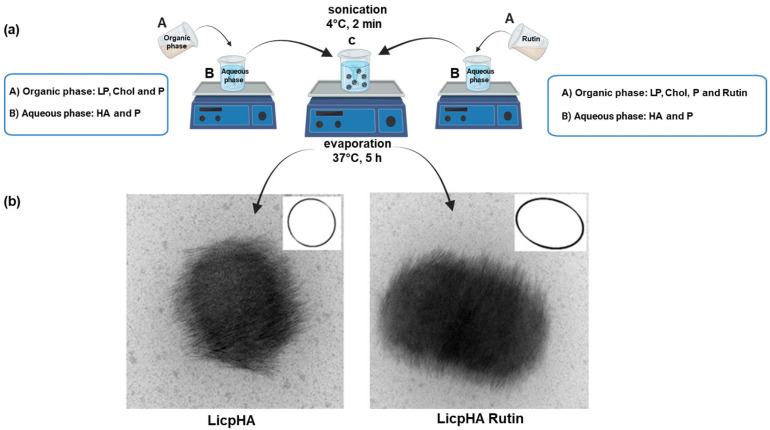
(**a**) Scheme of the preparation of LicpHA (**left**) or LicpHA Rutin (**right**). (**b**) Morphology of LicpHA and LicpHA Rutin according to TEM images (scale bar, 200 nm) (in the square: circularity measured via image analysis).

LicpHA Rutin was prepared using a modified nanoprecipitation technique using HA, P, Chol, and LP. Briefly, Rutin (20 mg), LP (300 mg), Chol (150 mg), and P (150 mg) were dissolved in 5 mL of ethanol to obtain a weight ratio LP:Chol:P of 2:1:1. The organic phase was added to 10 mL of aqueous phase containing HA (30 mg) in 0.05% (*w*/*v*) poloxamer water solution by vortexing (TechnoKartell, Milan, Italy) for 5 min. The resulting suspension was sonicated at 50% ultrasound for 2 min at 4 °C using a probe sonicator (Bioblock Vibracell, Fisher Bioblock Scientific, Illkirch, France) and stirred overnight until complete ethanol evaporation. Finally, the LicpHA Rutin dispersion was extruded three times through a regenerated cellulose syringe filter (pore size: 0.45 μm, filter size: 25 mm AlfaTech, Genova, Italy) and stored at 4 °C. LicpHA were prepared as described above but without the addition of Rutin (Figure 1). The reproducibility of the preparation method was evaluated by preparing the formulations three times. The nanoparticles LicpHA and LicpHA Rutin were not isolated but were used as dispersions in all experiments.

### 2.3. TEM Analyses

LicpHA morphology was studied through transmission electron microscopy (TEM, FEI Tecnai G12 Spirit Twin), equipped with a LaB 6 source and a FEI Eagle 4k CCD camera (Eindhoven, The Netherlands). The voltage acceleration was set at 120 kV. The sample was prepared by spraying 100 μL of ultra-diluted LicpHA in water onto a copper TEM grid (300 meshes, 3 mm diameter). The circularity of LicpHA was estimated using Image J software (Image J, 1.47; NIH, Bethesda, Rockville, MD, USA) on 10 randomly selected images at the same magnification (30,000×). Results were reported as mean ± standard deviation (SD). As for the circularity, a dedicated plugin was used, able to identify the boundaries of the selected objects and to calculate the circularity as follows:

C = 4π(A/P2)

where A is the area and P is the nanoparticle perimeter. A C value of 1.0 indicates a perfect circle.

### 2.4. Particle Size and Zeta Potential Measurements

The mean diameter and size distribution of LicpHA and LicpHA Rutin were measured by photon correlation spectroscopy (PCS) using a Coulter N5 (Beckman Coulter, Miami, FL, USA). All samples (100 µL LicpHA and LicpHA Rutin) were diluted in filtered Milli-Q water (0.22 µm pore size, polycarbonate filters, MF-Millipore, Microglass Heim, Italy) and analysed with the detector at 90°. The polydispersity index (PDI) was used to evaluate the particle size distribution. Zeta potential was measured with a Zetasizer Nano ZS (Malvern Instruments, Malvern, UK) on a 0.1 mg/mL LicpHA and LicpHA Rutin suspended in Milli-Q water at room temperature. The results were shown as the mean ± standard deviation (SD) of three measures (*n* = 3).

### 2.5. Stability Study

The physical stability of LicpHA and LicpHA Rutin was studied in terms of the trend of the hydrodynamic diameter when stored for 30 days at 4 °C and in the conditions of cytotoxicity experiments (at 37 °C for 72 h in RPMI-1640 supplemented with 10% FBS). The data analysed were the mean values from three independent experiments.

### 2.6. Differential Scanning Calorimetry (DSC) Analysis

The thermal analysis of LicpHA, LicpHA Rutin, and Free-Rutin (1.5–2 mg) was conducted by differential scanning calorimetry (DSC, Discovery DSC, TA Instruments, New Castle, DE, USA), running a ramp from −20 °C to 300 °C, with a heating rate of 10 °C/min. Before the test, the dispersion of nanoparticles was preliminarily concentrated and then gently dried under a hood overnight to remove the excess liquid phase.

### 2.7. Determination of the Total Amount of Rutin Content and the Encapsulation Efficiency

The total amount of Rutin in the LicpHA Rutin was measured spectrophotometrically. Briefly, 100 μL of LicpHA Rutin was mixed with 9.90 mL of DMSO and gently stirred for 30 min at 37 °C to dissolve the particles so that all the Rutin could be dissolved in the medium. The obtained solution was sonicated for 2 min at 35 Hz, and Rutin was quantified by a spectrophotometric assay (UV-1800; Shimadzu Laboratory World, Tokyo, Japan) at λ = 358 nm [33,34,35]. The interference at 258 nm of different excipients (PC, Col, P, and HA) on Rutin quantification was investigated. The linearity of the response was verified over the 0.2–50 μg/mL concentration range (R^2^ > 0.999). The total Rutin in LicpHA Rutin was calculated and reported as a concentration (mg/mL) and as a percentage concerning the Rutin used in the preparation [36]. Furthermore, encapsulation efficiency was determined via the indirect method of quantifying the concentration of Rutin in the supernatant. LicpHA Rutin (1 mL) was ultrafiltrated with an Amicon Ultra-15 centrifugal tube (regenerated cellulose membrane, 30.000 Nominal Molecular Weight Limit) at 4000 rpm for 20 min at 4 °C (Eppendorf Centrifuge 5702 R, Hamburg, Germany). Then, 100 μL of the supernatant was diluted with 4.9 mL of DMSO and sonicated in an ultrasonic bath for 2 min. The non-encapsulated Rutin was measured using the spectrophotometric assay described above. The lack of Rutin adsorption on the filter membrane was confirmed. The encapsulation efficiency (EE) was calculated [36]. The mean values were calculated from three independent batches.

### 2.8. Release Kinetics of Rutin

The in vitro release of Rutin from LicpHA Rutin and Free-Rutin was examined using cellulosic dialysis membrane tubing (Spectra-Por^®^ Float-A-Lyzer^®^ G2, cutoff 500–1000 Da, Sigma-Aldrich, Milan, Italy) in a shaker incubator (Argo Lab, SKI 4, VWR, Milan, Italy). Briefly, 4 mL of LicpHA Rutin and Free-Rutin (5.6 mg) were placed into the dialysis membrane tubing, which was then transferred into a transparent bottle containing 40 mL of PBS at pH 7.4. The samples were incubated at 37 °C ± 0.5 °C with shaking at 100 rpm for 96 h. At designated time points (0–96 h), 1 mL of the release medium was sampled and replaced with an equivalent volume of fresh dissolution medium. The concentration of Rutin released from LicpHA Rutin and Free-Rutin was determined by UV spectroscopy at λ = 364 nm. Concentrations of the analyte were determined by interpolating the standard curve. The demonstrated linearity was within the concentration range (y = 0.0151x + 0.0021 R^2^ = 0.9998) of 3–100 μg/mL. The cumulative amount of Rutin released over time was subsequently calculated. The results were obtained in triplicate and expressed as the mean ± standard deviation (SD).

### 2.9. Cell Culture and Treatments

Human umbilical vein endothelial cells, or HUVECs, were bought from ATCC, Manassas, VA, USA. The cells were cultured in a 100 mm Petri plate covered with 1% gelatin and filled with ECM medium (Sciencell, Carlsbad, CA, USA) containing 2% *v*/*v* of heat-inactivated FCS and gentamicin at 50 μg/mL. Endothelial toxicity was assessed through the following treatment protocol: endothelial cells were plated in a 96-well plate (200,000 cells/well) for 24 h. After three washes in PBS (pH 7.3), cells were unexposed to any drug (control group), treated for 24 h with epirubicin alone, or with 1 and 10 µM Free-Rutin, LicpHA, and LicpHA Rutin for 24 h. Briefly, a stock solution of epirubicin was made in dimethylsulfoxide (DMSO) to 5 mM and properly stored at –20 °C; after, it was diluted directly in a cell culture medium up to the final concentration from 10 to 1000 nM. Free-Rutin was pre-dissolved in absolute ethanol (Sigma Aldrich, Milan, Italy) as a stock solution and subsequently diluted directly in the cell culture medium up to the desired concentrations (1 and 10 µM); LicpHA and LicpHA Rutin were instead directly diluted in the HUVEC culture medium and then placed in contact with the cells at the final concentrations of the payload (Rutin) of 1 or 10 µM. Then, the cells were subjected to cell viability and inflammatory tests as described below.

### 2.10. Cellular Uptake Studies

For the uptake quantification study of LicpHA, a protocol described in our previous work [37] was followed. LicpHA were prepared by incorporating 1 mg of fluorescent Nile Red (NR) into the organic phase, resulting in LicpHAF. LicpHAF was subjected to centrifugation at 10,000 rpm for 10 min and washed three times to eliminate any non-encapsulated NR. In brief, LicpHAF were dispersed in a cell culture of HUVEC cells incubated for a time ranging from 0.5 to 24 h. After the incubation periods, the supernatant was removed and cells were washed three times with 10 mM PBS and then with the lysate with 0.1 mL of 0.5% Triton X-100 in 0.2 N NaOH. The fluorescence of the cell lysate (λ_exc_ = 555 nm, λ_em_ = 580 nm) allowed the evaluation and quantification of the membrane-bound and internalized LicpHAF, using a calibration curve dispersed in a cell lysate solution (untreated cells dissolved in 1 mL of the Triton X-100/0.2 N NaOH solution). For both the calibration curve and LicpHAF cellular uptake determination, the fluorescence was measured at the proper wavelengths using a spectrofluorometer (xMark Microplate spectrofluorometer; Bio-Rad Laboratories, Milan, Italy).

### 2.11. Assessment of Cell Survival and LDH Release

A modified MTT technique known as the MTS assay was used to analyse the viability of the cells. The -2H-tetrazolium inner salt (MTS) test yields a water-soluble formazan product with a maximum absorbance at 450–500 nm in the phosphate-buffered saline solution when the tetrazolium compound, MTS, is combined with phenazine methosulfate. For cell survival experiments, HUVECs were left unexposed (control) or exposed to epirubicin (10 to 1000 nM) alone or in combination with LicpHA, LicpHA Rutin (at 1 or 10 µM of Rutin), or Free-Rutin (1 or 10 µM). Following the protocol outlined in the literature, the treated endothelial cells were cultured with 100 μL of an MTS solution (at 0.5 mg/mL) for 4 h at room temperature [38]. The cells were then rinsed three times with PBS at a pH of 7.4. Using I-control 5.0 software, absorbance measurements were obtained at 450 nm using a Tecan Infinite M200 platereader (Tecan Life Sciences Home, Männedorf, Switzerland). The following formula (Equation (1)) was used to obtain the relative cell viability (%):

(1) $$Relative\;cell\;viability\left(\%\right)=\frac{\left(A\right)test}{\left(A\right)control}\times 100$$

where “(A)test” is the test sample absorbance and “(A)control” is the absorbance of untreated cells.

Using the Pierce Micro BCA protein assay kit (Thermo Fisher, Milan, Italy), the total protein content was determined after assessing cell cytotoxicity [39]. Briefly, after the cells were washed with ice-cold PBS, they were treated for 15 min in 150 μL of cell lysis buffer (0.5% *v*/*v* Triton X100) + 150 μL of Micro BCA protein assay kit. The absorbance at 562 nm was measured on a plate reader. HUVECs were treated with epirubicin (at 200 nM, around the IC_50_ value) alone, in combination with LicpHA, LicpHA Rutin (at 1 or 10 µM of Rutin), or Free-Rutin (1 or 10 µM) for the LDH experiment. A Cytotoxicity Detection Kit (LDH) from Roche Applied Science was used to measure the amount of LDH released into the supernatant by injured cells. A microplate spectrofluorometer was used to measure the signals at 490 nm to quantify LDH.

### 2.12. NLRP-3 and MyD-88 Expression Studies

Endothelial cells were lysed through a lysis buffer composed of Tris-HCl 50 mM, PMSF 1 mM, EDTA 1 mM, NaF 20 mM, Na_3_VO_4_ 3 mM, NaCl 100 mM, and protease inhibitors. Lysates were centrifuged supernatants and were used for the quantification of NLRP-3 through an NLRP-3 ELISA Kit (code OKEH03368, Aviva Systems Biology, San Diego, CA, USA) and MyD-88 through a MyD-88 ELISA Kit (code ab171341, Abcam, Milan, Italy). The human NLRP-3 ELISA test had a sensitivity of less than 0.078 ng/mL and a detection range of 0.156–10 ng/mL. The human MyD-88 ELISA had a sensitivity of less than 10 pg/mL and a detection range of 156 pg/mL–10,000 pg/mL.

### 2.13. Cytokines, Chemokines, and Growth Factors Assay

Cytokines, chemokines, and growth factors with pro-inflammatory and anti-inflammatory effects (IL1β, IL4, IL6, IL-17α, IL10, and TNFα) were quantified in endothelial cells using selective anti-human ELISA kits, following the manufacturer’s instructions in line with other work [33]. Briefly, after the treatments described in Section 2.9, the cell supernatants were centrifuged at 1500 rpm and analysed for cytokine quantification through selective ELISA kits; the results were characterized by a sensitivity below 5.6 (pg/mL) and a detection range of 1–32,000 pg/mL.

### 2.14. Statistical Analysis

The statistical data were analysed using GraphPad Prism 9.5.1 software (GraphPad Software, Inc., San Diego, CA, USA). A two-sample unpaired *t*-test was conducted to assess the statistical distinction between the two treatment groups. Tukey’s multiple comparison tests followed a one-way ANOVA analysis of variance test in case of multiple comparisons. The *p* < 0.05 was considered significant.

## 3. Results and Discussion

### 3.1. Preparation of Hybrid Nanoparticles

LicpHA and LicpHA Rutin were prepared using a modified nanoprecipitation technique that allowed the self-assembling of HA, LP, Chol, and P in one step. In contrast to the traditional nanoprecipitation method, where the drug and polymer are placed in an organic solvent and the lipid in water [19], LP, Chol, and Rutin were dissolved in ethanol and HA in water; P was added to both phases. Phosphatidylcholine was used as a basic component of nanoparticles due to its biocompatibility, with a structure strengthened by the addition of Chol and P. The use of a 1:1 Chol:P weight ratio was used to obtain rigid particles [40]. Moreover, P forms a multilamellar cohybrid structure with HA, increasing its structural strength and drug-loading capacity [22,30]. The presence of HA in the nanoparticles was verified by FTIR analyses on LicpHA Rutin formulation (Figure S1). Ultimately, the work used a method for preparing hybrid nanoparticles of applying a simple, sustainable procedure that includes novel, greener alternatives [41].

### 3.2. Physicochemical Characterisation of the Hybrid Nanoparticles

TEM images of LicpHA are shown in Figure 1b. They showed a non-spherical shape with a circularity of 0.782 ± 0.140. It was possible to distinguish the presence of regular structures on the surface that can be associated to the crystalline state of lipid phases, in addition to other phases, including HA, that physically interact with phospholipids, as reported in previous works [22,30]. In this case, the presence of Rutin was confirmed by zeta potential measurements (Table 1) which showed a decrease in the surface charge from −35.1 ± 1.2 mV to −30 ± 0.5 mV (*p* < 0.05), moving from LicpHA to LicpHA Rutin systems, respectively. This could be ascribable to the physical interactions of Rutin with phospholipids, in agreement with previous experimental evidence on similar systems [42]. Notably, zeta potential values more negative than −30 mV generally tend to promote repulsive phenomena among charged particles, thus guaranteeing optimal nanoparticle stability in dispersions [43]. This concurs with preventing nanoparticle aggregation and binding to plasma proteins, thus minimising the risk of vessel occlusion after the in vivo administration of nanoparticles [22].

LicpHA tended to form a heterogeneous population with an average diameter of 179 ± 4 nm (Table 1), with an increase in the mean value (209 ± 5 nm) for LicpHA Rutin due to the local reorganisation of the nanoparticle structure after the drug molecule intercalation, in agreement with previous experimental shreds of evidence [22,44]. The polydispersity index (PDI) values obtained indicated a heterogeneous population (Table 1); these values are generally related to HA with a high molecular weight, which increases the viscosity of the solution [24,43]. Moreover, the laser measurement may have been strongly influenced by the shape factor of the nanoparticles as verified by TEM analysis.

**Figure 2:**
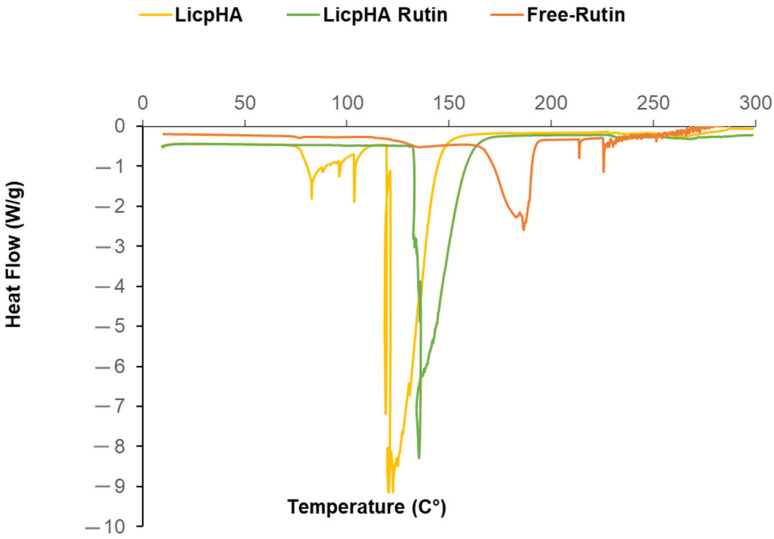
DSC thermograms of Free-Rutin, LicpHA, and LicpHA Rutin. The results were obtained from at least three independent experiments.

Thermal studies of LicpHA, Free-Rutin, and LicpHA Rutin are reported in Figure 2. The Free-Rutin and LicpHA showed endothermic peaks at 187.29 °C and 122.75 °C, respectively, relating to the fusion mechanisms. In the case of LicpHA Rutin, the melting peak associated with Rutin was not recognized, while the fusion temperature was 135.28 °C. This shift in the endothermic peak could be due to the displacement of water molecules due to the Rutin loading in the nanoparticles [22].

The total amount of Rutin and the encapsulation efficiency of LicpHA Rutin were estimated. No interference from excipients was detected, as also reported in the literature [45,46,47]. The total amount of Rutin in the LicpHA Rutin dispersion was 1.33 ± 0.13 mg/mL, corresponding to 68.9% of the drug used for the preparation. The remaining part of Rutin was lost during the extrusion process due to the high viscosity of the dispersion. The encapsulation efficiency of Rutin in the LicpHA Rutin was 45 ± 1% (Table 1). Moreover, the stability of LicpHA and LicpHA Rutin was demonstrated in both water for 30 days and in cell culture medium for 72 h (Table 2). The sizes of LicpHA and LicpHA Rutin were analysed in water for 30 days (Table 2). The mean diameter of both formulations did not change significantly over time (*p* > 0.05), indicating the good physical stability of the nanoparticles in the dispersion, as in agreement with the zeta potential results. Furthermore, no significant variation in the size of LicpHA and LicpHA Rutin was observed after dilution in the RPMI-1640 cell medium and storage at 37 °C for 72 h, suggesting that there was no interaction between the nanoparticles and the serum proteins in the medium during the in vitro cell studies. The fact that these nanoparticles maintain their stable diameter for up to 72 h suggests that they have a hydrophilic surface, which likely leads to weaker interactions with serum proteins [48]. This information suggests potential applications in drug delivery or other biomedical fields, where stable nanoparticles with hydrophilic surfaces can offer advantages such as a prolonged circulation time or reduced interactions with serum proteins, thereby potentially improving efficacy and safety profiles [48].

### 3.3. Release Kinects

**Figure 3:**
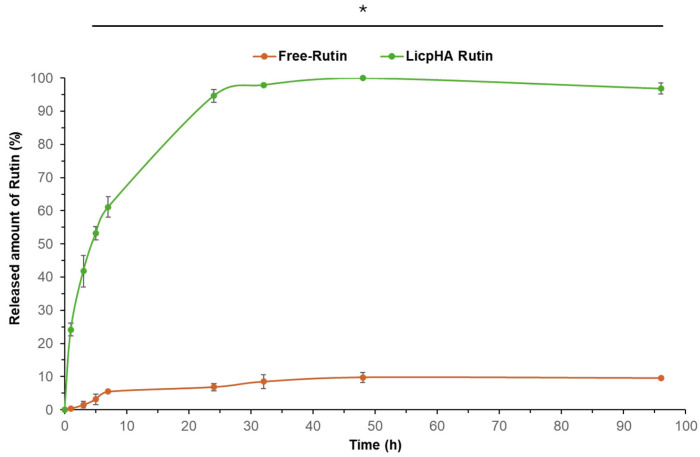
In vitro release profiles of Free-Rutin and Rutin from LicpHA Rutin in PBS at 37 °C. * *p* < 0.00001 of Free-Rutin to LicpHA Rutin.

In vitro experiments were conducted to analyse the release profile of Rutin. Specifically, release studies were conducted on both the LicpHA Rutin and Free-Rutin, with the results illustrated in Figure 3. Free-Rutin completely passed through the membrane within 24 h [49]. The LicpHA Rutin displayed an appealing release profile, sustaining the drug release over a period of 96 h. A slight burst release (around 20%) was observed during the first 3 h of the experiment, suggesting a small quantity of drugs present on the surface of LicpHA Rutin and indicating a uniform distribution of Rutin within the formulation. The release exhibited a sustained pattern with 61% released at 7 h, escalating to 94% after 24 h, and then transitioning to a slower release phase. Complete drug release was achieved within approximately 30 h. The predominant mechanism of drug release observed could be diffusive, according to findings in the literature [50].

### 3.4. Cellular Uptake Study

**Figure 4:**
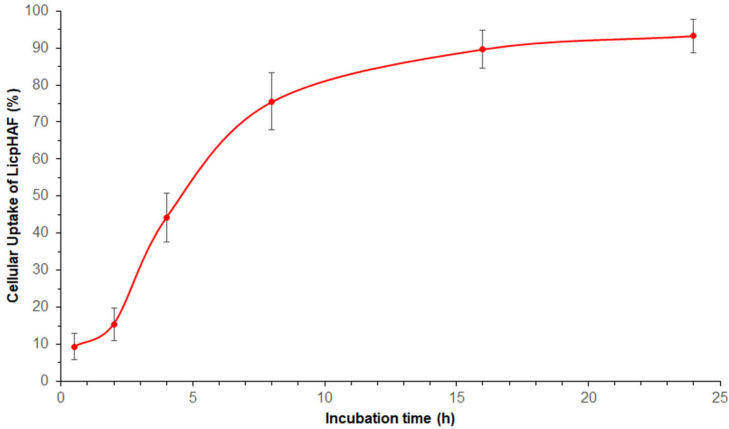
Overall cellular uptake (%) of LicpHAF in HUVEC cells (5 × 10^3^ cells/well) from 0.5 to 24 h.

As shown in Figure 4, the uptake of LicpHAF was time-dependent in HUVEC cells. Notably, after 2 h of incubation, approximately 15.4 ± 4.4% of LicpHAF were internalized; after 8 h of incubation, the calculated uptake of nanoparticles increased significantly to a 75.5 ± 7.7%. A plateau phase was seen between 8 and 24 h of incubation with a maximum value 93.3 ± 4.5% at 24 h. The cellular uptake exhibited a behaviour typical of receptor-mediated endocytosis, in line with the literature on HA-based nanoparticles. Consistent with the existing literature, it was plausible that LicpHA interacted with human cells, including HUVECs, through CD44-mediated endocytosis [51].

### 3.5. Cell Viability and LDH Release of Endothelial Cells

**Figure 5:**
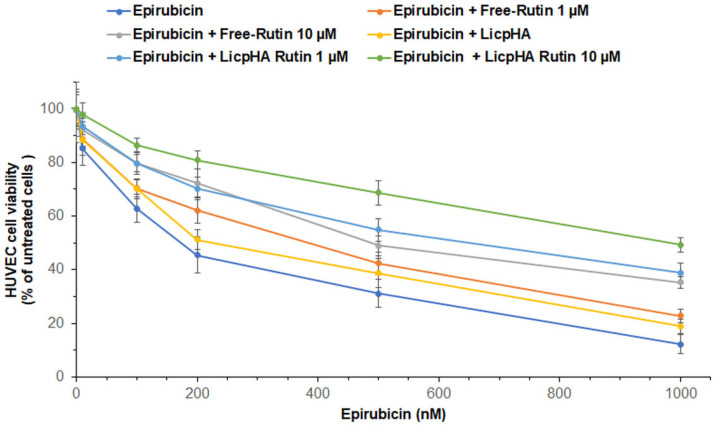
Cell viability in human endothelial cells after 24 h of incubation with epirubicin, unformulated Rutin at 1 and 10 µM, LicpHA Rutin or LicpHA Rutin at 1 and 10 µM alone or combined with epirubicin. The error bars depict means ± SD.

Considering the clinically relevant endothelial pro-inflammatory phenotype induced by anthracycline therapy in cancer patients, cytoprotective studies were performed on endothelial cells (HUVECs cell line, an in vitro model of several endothelial barriers) exposed to LicpHA Rutin combined with epirubicin [52,53,54]. Cell viability studies demonstrated that epirubicin exerts a vasculotoxic effect in a concentration-dependent manner with an IC_50_ value of ± 150 nM (Figure 5). Epirubicin combined with LicpHA did not change the vasculotoxic profile of epirubicin alone. Therefore, LicpHA did not exert cytoprotective effects. Notably, when associated with Free-Rutin and the LicpHA Rutin, at 1 and 10 µM, human endothelial cells increased their viability during exposure to epirubicin (Figure 5). Specifically, the best vasculoprotective profile was seen in epirubicin + LicpHA Rutin at 10 µM with an IC_50_-epirubicin-related value of around 900–1000 nM. These results indicated the vasculoprotective properties of LicpHA Rutin.

**Figure 6:**
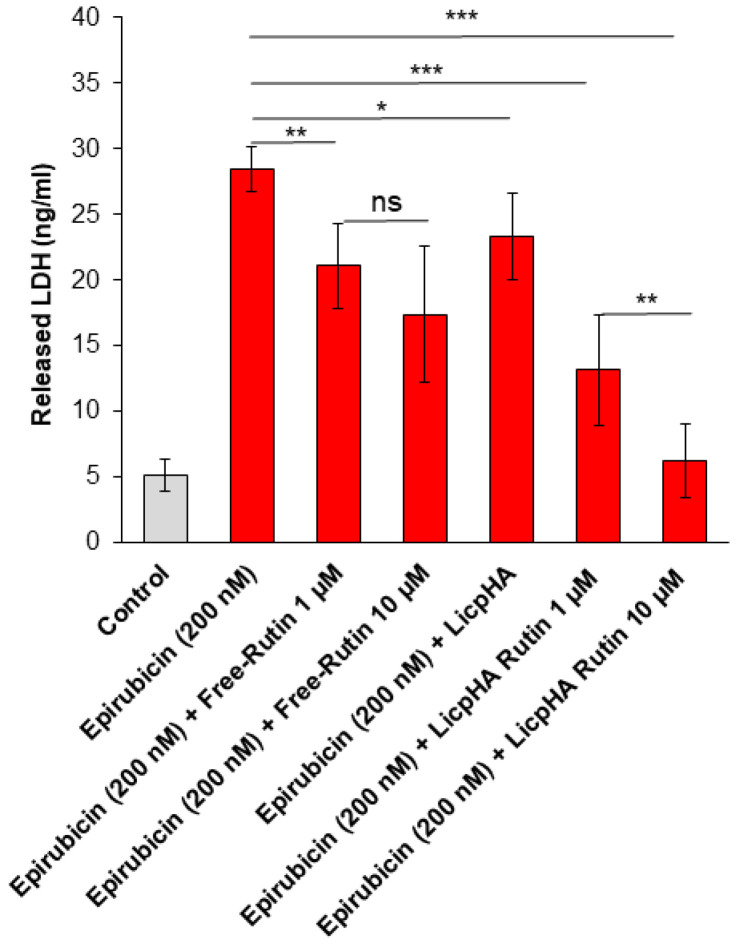
LDH release (ng/mL) by human endothelial cells after 24 h of incubation with epirubicin, Free-Rutin at 1 and 10 µM, LicpHA or LicpHA Rutin at 1 and 10 µM alone or combined with epirubicin. Error bars depict means ± SD. *p*-values for the indicated compounds relative to untreated cells are *** *p* < 0.001. ** *p* < 0.01. * *p* < 0.05. ns, not significant.

Similarly, LDH release was significantly increased after incubation with epirubicin (Figure 6). The data showed that LDH release from endothelial cells decreased with an increasing LicpHA Rutin concentration when compared to epirubicin-treated cells. Notably, Free-Rutin is easily degraded by the external environment (i.e., oxidized by the culture medium and body proteins). Its cellular intake in cell cytoplasm is difficult without structural changes that could limit its antioxidant and anti-inflammatory activities [55,56]. Based on these results, LicpHA Rutin improved the Rutin-related cytoprotective effects in HUVEC cells exposed to toxic drugs.

#### 3.5.1. NLRP-3 and Myd-88 Expression in Endothelial Cells

**Figure 7:**
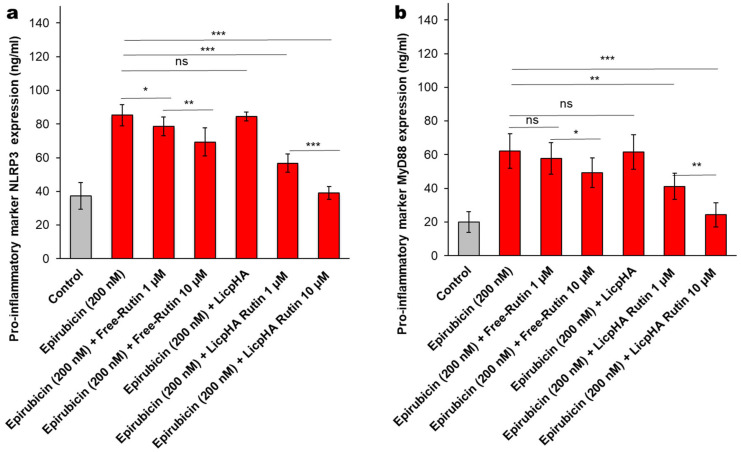
(**a**) NLRP-3 and (**b**) MyD-88 expression (ng/mL) in human endothelial cells incubated with epirubicin, Free-Rutin at 1 and 10 µM, LicpHA or LicpHA Rutin at 1 and 10 µM alone or combined with epirubicin. The error bars depict means ± SD. *p*-values for the indicated compounds relative to untreated cells are *** *p* < 0.001. ** *p* < 0.01. * *p* < 0.05. ns, not significant.

The NLRP-3 inflammasome and Myd-88 myddosome are key players of the cytokine storm involved in endothelial damages and neurotoxicity [57]. NLRP3 expression was increased after exposure to epirubicin when compared to untreated cells (Figure 7a); lower levels of NLRP3 after co-incubation with Free-Rutin and LicpHA Rutin were seen. LicpHA Rutin exerts the best anti-inflammatory effects by reducing up to 60% of the expression of NLRP3 when compared to epirubicin-treated cells. Similar behaviour was seen for MyD88 myddosome; Myd88 expression is enhanced after exposure to epirubicin when compared to untreated cells (Figure 7b). Lower levels of Myd88 after co-incubation with Free-Rutin and LicpHA Rutin were also seen, indicating the involvement of these pathways in the vascular protection of Rutin during epirubicin exposure.

#### 3.5.2. Cytokines and Chemokines Levels in Endothelial Cells

**Figure 8:**
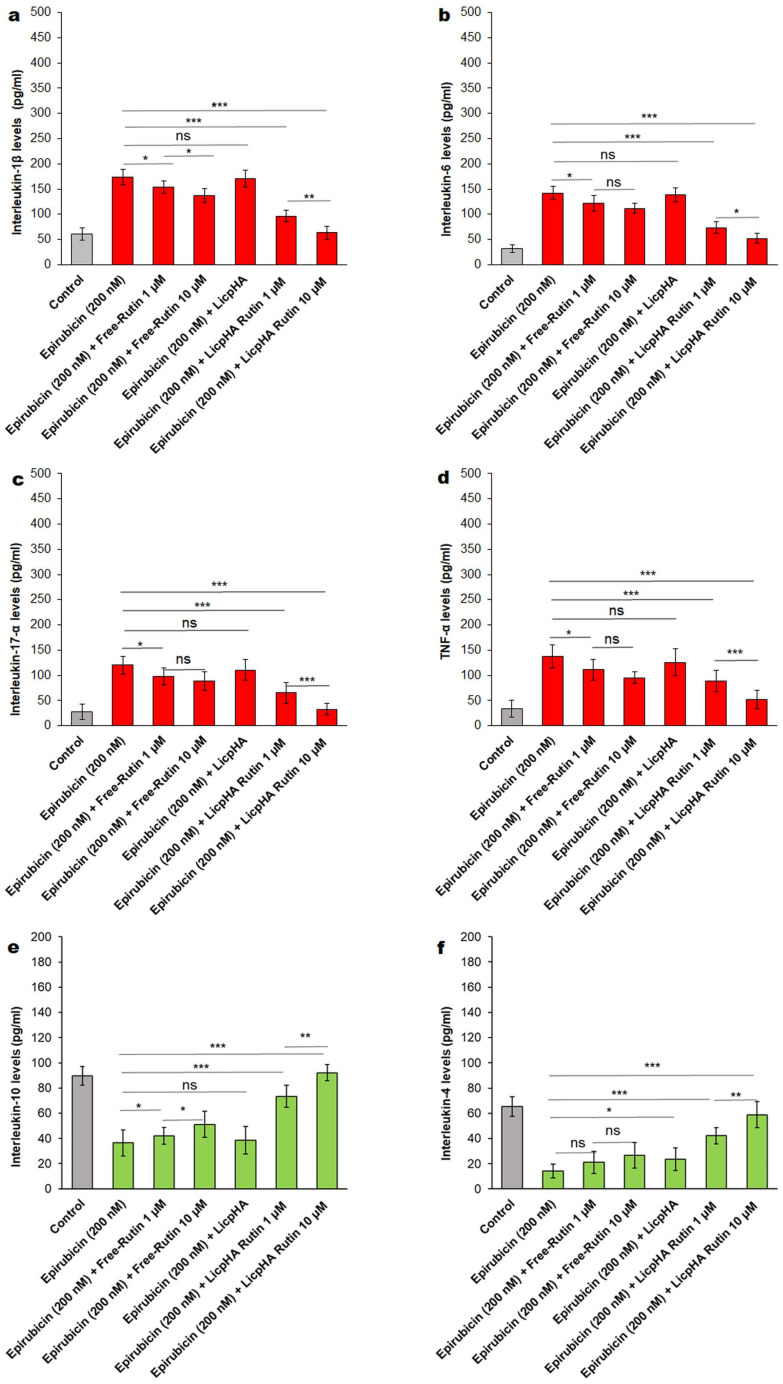
(**a**) IL-1β, (**b**) IL-6, (**c**) IL17-α, (**d**) TNF-α, (**e**) IL-10, and (**f**) IL-4 expression (pg/mL) in human endothelial cells incubated with epirubicin, Free-Rutin at 1 and 10 µM, LicpHA or LicpHA Rutin at 1 and 10 µM alone or combined with epirubicin. The error bars depict the means ± SD. *p*-values for the indicated compounds relative to untreated cells are *** *p* < 0.001. ** *p* < 0.01. * *p* < 0.05. ns, not significant.

Considering that NLRP3 and MyD88 are key players in anthracycline-mediated neurotoxicity, sarcopenia, cardiotoxicity, and vascular toxicity [33,57], it was investigated in terms of the synthesis of several anti-inflammatory and pro-inflammatory cytokines involved in the vascular toxicity of epirubicin. As clearly described in Figure 8a–f, epirubicin treatment increased cytokines and chemokines with a pro-inflammatory activity, which is in line with the literature. In contrast, the LicpHA Rutin was able to change the cellular microenvironment, increasing the anti-inflammatory effect on the endothelial phenotype. For example, when compared with untreated cells, HUVECs exposed to epirubicin had increased IL-1β and IL-6 levels (Figure 8a,b) (*p* < 0.001 vs. control); in contrast, the level of IL-10 (an anti-inflammatory cytokine) strongly decreased when compared to that in untreated cells (*p* < 0.001 vs. control) (Figure 8e). After coincubation with Free-Rutin and the LicpHA Rutin, the rates of increase in the levels of these cytokines were significantly reduced, indicating that the anti-inflammatory effect of Rutin agreed with other preclinical studies. The greatest anti-inflammatory effects were observed for LicpHA Rutin, in which the IL-1β and IL-6 levels were similar to the baseline values in untreated cells (Figure 8a,b). Data of the present study, although in vitro, indicated that LicpHA Rutin induced an anti-inflammatory phenotype through the reduction of NLRP3- and Myd-88-derived cytokines secreted by endothelial cells exposed to a clinically relevant concentration of epirubicin.

A key point of LicpHA Rutin was, therefore, the improvement of the biological activities of Rutin when compared to those of Free-Rutin as follows: Rutin is easily degraded by the external environment, i.e., oxidized by the culture medium and by body proteins, and its cellular intake in the cell cytoplasm is complex without structural changes which could limit its antioxidant and anti-inflammatory activities [58]. LicpHA Rutin protected the external environment (pro-oxidant) and promoted a greater cellular intake ability of Rutin, consequently improving its cytoprotective properties.

## 4. Conclusions

In this work, LicpHA Rutin was proposed as a therapeutic agent for anthracycline-induced endothelial damages. Due to their physical interactions, a nanosized structure able to load 45% of Rutin was obtained with a peculiar morphology and chemical-physical properties. When compared to Free-Rutin, LicpHA Rutin released Rutin in vitro in a controlled manner and 96% was recovered in the dissolution media after 30 h. Cellular uptake studies clearly demonstrated that LicpHA could interact with human cells with a behaviour typical of receptor-mediated endocytosis. LicpHA Rutin has been shown to exert vasculo-protective effects on endothelial cells exposed to clinically relevant epirubicin doses through NLRP3- and MyD-88-related pathways, reducing intracellular IL-1β and IL-6 levels. In summary, Rutin-loaded nanoparticles could be proposed as an innovative pharmacological tool to prevent vascular damages induced by anthracyclines in cancer patients.

## Figures and Tables

**Table 1:** Summary of the main properties of LicpHA and LicpHA Rutin: Size, PDI, zeta potential, total amount of Rutin (%), and encapsulation efficiency (%). The results are reported as the mean ± SD (*n* = 3). * *p* < 0.05.

Formulation	Mean Diameter (nm)	PDI	Zeta Potential (mV)	Total Amount of Rutin (%)	Encapsulation Efficiency (%)
LicpHA	179 ± 4 *	1.0 ± 0.0	−35 ± 1 *	-	-
LicpHA Rutin	209 ± 5 *	1.1 ± 0.0	−30 ± 1 *	69 ± 2	45 ± 1

**Table 2:** Mean diameter of LicpHA and LicpHA Rutin dispersions stored at 4 °C for 30 days and after dilution in RPMI-1640 medium at 37 °C for 3 days.

Formulation	4 °C			RPMI 37 °C			
	Day 0	Day 15	Day 30	Day 0	Day 1	Day 2	Day 3
LicpHA	179 ± 4	199 ± 3	190 ± 3	186 ± 3	182 ± 3	194 ± 2	189 ± 2
LicpHA Rutin	209 ± 5	209 ± 2	206 ±2	202 ± 1	202 ± 1	208 ± 3	210 ± 0

## Data Availability

The data presented in this study are available on request from the corresponding author.

## Supplementary Materials

The following supporting information can be downloaded at: https://www.mdpi.com/article/10.3390/pharmaceutics16080985/s1, Figure S1: FTIR analyses: spectra of LicpHA Rutin, HA and LP. References [59,60] are cited in the Supplementary Materials.
