# Supplementary material for: Hyaluronic Acid-Based Nanoparticles Loaded with Rutin as Vasculo-Protective Tools against Anthracycline-Induced Endothelial Damages
Source: Pharmaceutics. 2024 Jul 25;16(8):985. doi: 10.3390/pharmaceutics16080985 (PMC11357640; doi:10.3390/pharmaceutics16080985)
Supplement: Supplementary file 1 [file pharmaceutics-16-00985-s001.zip › pharmaceutics-3042893-supplementary.pdf]

Method

Fourier Transform Infrared Spectroscopy (FTIR) analysis

FTIR spectra for pure materials (LP and HA) and LicpHA Rutin were measured using a Shimadzu 8400S spectrophotometer with OPUS 6.0 software (Shimadzu, Billerica, MA, USA). Briefly, an appropriate quantity of KBr and the samples (at a ratio of 100:1) were mixed by grinding in an agate mortar. Pellets were then formed from approximately 100 mg of the mixture. The spectra were recorded across the range of 4500–250 cm^{-1}.

Results

The spectrum of LicpHA Rutin (Figure S1) revealed characteristic peaks of HA at 3443 cm-1, indicative of broad hydroxyl group stretching, at 1720 cm^{-1} and 1648 cm^{-1}, corresponding to the carbonyl stretching bands of carboxylic acids and amides, respectively, and at 1151 cm^{-1} and 1034 cm^{-1}, related to ether bands [1]. Thus, the presence of HA in LicpHA Rutin was assessed.
Moreover, distinct peaks associated with the lipid phase were also evident, such as the asymmetric (2916 cm^{-1}) and symmetric (2850 cm^{-1}) stretching modes of CH_2 groups, and the stretching mode of the C=O group at 1740 cm-1 [2].

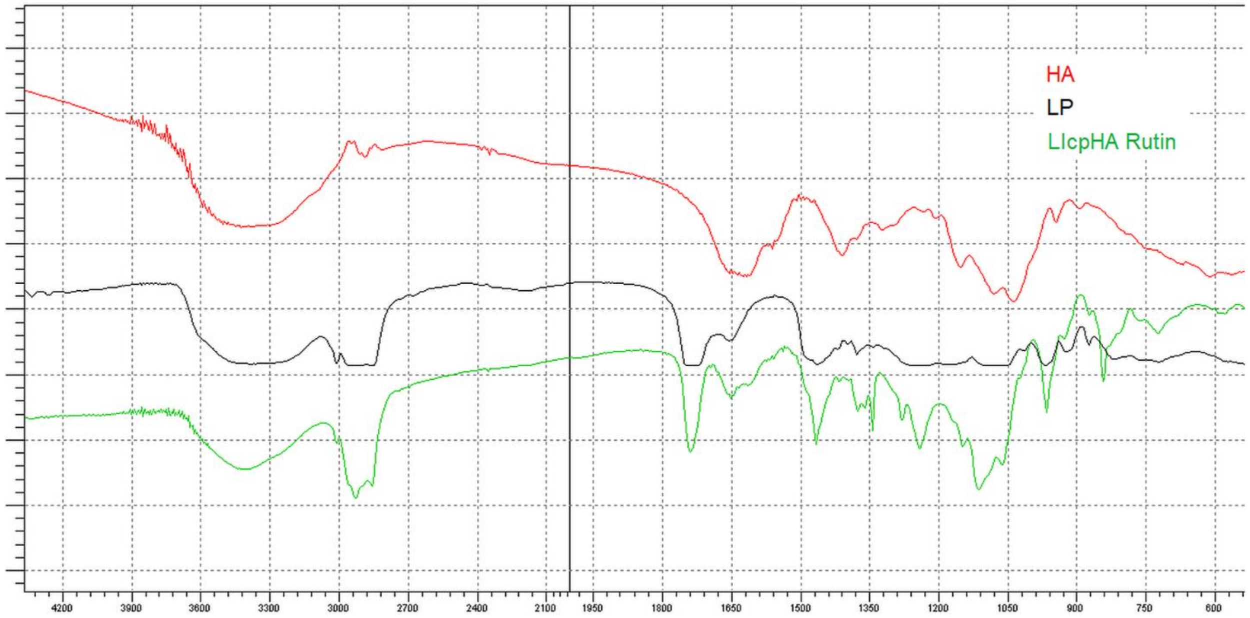

Figure S1. FTIR analyses: spectra of LicpHA Rutin, HA and LP.

References

[1] Carneiro, J.; Döll-Boscardin, P.M.; Fiorin, B.C.; Nadal, J.M.; Farago, P.V.; Paula, J.P.D. Development and Characterization of Hyaluronic Acid-Lysine Nanoparticles with Potential as Innovative Dermal Filling. Braz. J. Pharm. Sci. 2016, 52, 645–651, doi:10.1590/s1984-82502016000400008.

[2] Portaccio, M.; Faramarzi, B.; Lepore, M. Probing Biochemical Differences in Lipid Components of Human Cells by Means of ATR-FTIR Spectroscopy. Biophysica 2023, 3, 524–538, doi:10.3390/biophysica3030035.
